# The Battle Against COVID-19 in Jordan: An Early Overview of the Jordanian Experience

**DOI:** 10.3389/fpubh.2020.00188

**Published:** 2020-05-07

**Authors:** Ala'a B. Al-Tammemi

**Affiliations:** Department of Epidemiology and Global Health, Umeå University, Umeå, Sweden

**Keywords:** COVID-19, Jordan, crises management, pandemic, decision makers

## Abstract

Since the initial spark of the COVID-19 outbreak in December 2019, which was later declared by the World Health Organization (WHO) to be a global pandemic, all affected countries are implementing various preventive and control measures to mitigate the spread of the disease. The newly emerging virus brings with it uncertainty—not only regarding its behavior and transmission dynamics but also regarding the current lack of approved antiviral therapy or vaccines—and this represents a major challenge for decision makers at various levels and sectors. This article aims to provide an early overview of the COVID-19 battle within the Jordanian context, including general reflections and conclusions on the value of collaborative efforts in crises management.

## Introduction

It has been over five decades since the first discovery of human coronaviruses (1). A series of outbreaks and epidemics of respiratory illnesses have been attributed to various types of these viruses, such as Severe Acute Respiratory Syndrome (SARS) and Middle East Respiratory Syndrome (MERS), which were caused by SARS-CoV and MERS-CoV, respectively, in addition to the current Coronavirus Disease 2019 (COVID-19) (1, 2). COVID-19 is caused by novel SARS-CoV-2, which, to a certain degree, possesses genomic similarities to MERS-CoV and SARS-CoV (2, 3).

These coronaviruses are transmitted from their animal origins to humans through an intermediate host, such as camels in the case of MERS and civet cats in the case of SARS (1, 4). Unfortunately, the intermediate host that is responsible for the interspecies (animal to human) transmission of the novel SARS-CoV-2 is still under debate (2); pangolins could be a potential candidate (4), however, it is still debatable whether the primary origin of the novel SARS-CoV-2 stems from bats or pangolins (2).

In late December 2019, pneumonia of an unknown cause was reported in Wuhan city, China, and, from this point of origin, the outbreak has spread extensively to a global scale (3). On the 30th of January 2020, the WHO declared the outbreak of COVID-19 as a global public health emergency, and, upon the exponential increase in the number of cases and countries affected by the disease, COVID-19 was then declared as a pandemic on the 11th of March 2020 (4–6).

The symptoms of COVID-19 mostly appear within 2–14 days of acquiring the virus, and a different range of symptoms and severity can affect patients, including fever, dry cough, dyspnea, sore throat, nausea, vomiting, diarrhea, myalgia, and fatigue (5, 7, 8). Although most COVID-19 patients develop a mild degree of symptoms and exhibit spontaneous recovery, there is still a proportion of patients, especially older age groups with underlying comorbidities, that are at higher risk of developing a more severe illness that is associated with complications (5, 7). As of the 16th of April 2020, 2:00 CEST, the WHO announced that 213 countries and territories have been affected by the COVID-19 with 1 995 983 confirmed cases and 131 037 confirmed deaths due to this disease (9).

COVID-19 has high transmissibility (10). The mechanism of the viral spread in COVID-19 still has some degree of uncertainty (5). However, human to human transmission is reported to occur via respiratory droplets and aerosols that result from infected persons as well as via direct contact with contaminated objects (3, 10).

Various preventive and control measures at different levels have been implemented in different countries around the world in order to combat the spread of COVID-19. Among these measures, on an individual level, are maintaining a social distance of at least 3 feet between individuals, washing hands frequently, using hand sanitizers, practicing coughing and sneezing etiquette, avoiding handshaking and kissing, avoiding direct contact with ill persons, especially those who exhibit symptoms of respiratory infections, and wearing face masks in certain situations (3, 5, 10–12).

As of the date of writing this article, most COVID-19 patients receive symptomatic and supportive treatment, but there is no definite antiviral therapy for COVID-19 yet (3, 10). The scientific community is currently working vigorously to develop an effective antiviral therapy as well as a vaccine for COVID-19 (3, 5, 10, 13).

## COVID-19 and the Jordanian Context

Jordan is located in the Eastern Mediterranean region with an estimated population of around 10.6 million inhabitants and a total area of 89,342 square kilometers (14, 15). Jordan shares borders with Iraq, Israel and the occupied Palestinian territory, Saudi Arabia, as well as Syria (15). The World Bank currently classifies Jordan as an upper middle-income country with a Gross National Income (GNI) of 9,430 international dollars per capita in 2018 (16). Since the beginning of the COVID-19 outbreak, the Jordanian government has followed the recommendations and updates provided by the WHO. A series of preventive and control strategies at the local and national levels have been implemented in order to limit the spread of COVID-19 inside Jordan.

The fight against COVID-19 in Jordan is led by the government through a collaborative multi-disciplinary team at the highest levels at the National Center for Security and Crises Management (NCSCM) (17, 18). This crisis task force is comprised of expert decision makers from different ministries, sectors, and organizations in order to reach for and provide the best evidence-based recommendations for implementation (18). Decisions regarding different life perspectives are cautiously and continuously updated and disseminated to the public through official authorities. In addition, teams of experts in epidemiological surveillance are currently working across the country to tackle cases and provide random viral testing and surveillance (12). Furthermore, the number of confirmed cases, recoveries, and deaths are publicly announced to the population each day through official reports by the government. Keeping up with the advancements in digital health, a COVID-19 website provided in the Arabic language has been created by a collaborative efforts between The Ministry of Health and The Ministry of Digital Economy and Entrepreneurship, and it aims to spread awareness, knowledge, statistics, and recommendations to the public (12). Besides, a collaboration between the Jordanian government and Facebook was developed to spread awareness about COVID-19 to Jordanians who access Facebook, as it is one of the most commonly used social networking sites among Jordanians (12).

Jordan is considered a touristic country and a main connection point for many flights and trips within the region, and this, along with the noticeable increase in number of COVID-19 cases globally, has meant that the government has started to implement (periodically revised) strict rules and measures relating to travel, education, religious and social events, as well as working within various industries (17).

The primary step in preventing and controlling the spread of COVID-19 in Jordan started at the country's entry ports through temperature screening of incoming travelers as well as enforcing a quarantine to those who came from countries with high COVID-19 spread (12, 17). The turning point in the country's preventive and control measures was dated as the 17th of March 2020 upon declaring the national defense law in order to mitigate the spread of COVID-19 in Jordan. On the 20th of March 2020, a decision for a nationwide curfew was declared with strict rules on individuals' mobility and extreme fines for violations (17). During the curfew, decisions are announced regularly, and the degree of restricting individuals' mobility varies during the week with oscillation between round-the-clock and partial curfew (17). The country's preventive and control measures are briefly highlighted and discussed in the following section.

## Preventive and Control Measures Against COVID-19 in Jordan

### Overview of Decisions on Travel Restrictions

The decisions about international travel have progressed through many stages that accompanied the growth in the number of COVID-19 cases globally, especially in countries that have been struck severely by the disease.

These measures started with banning the entry of incoming non-Jordanian travelers from specific countries, including China, South Korea, Italy, and Iran, with exceptions given to Jordanian nationals who were allowed to enter Jordan with an obligatory 14 days of quarantine at specified facilities provided that were regulated by the government (12, 17). Later on, more countries have been added to the ban and restriction list (12, 17). The most extreme measure was in announcing a total country lockdown starting effectively from the 17th of March 2020 until further notice; the only exception was in cases of commercial cargo movements (17).

### Overview of Decisions Regarding Religious and Social Events

The Jordanian population is characterized by high levels of sociability and social events that occur on a daily basis with handshaking as a traditional and essential form of greeting. Keeping that in mind, these societal characteristics make it somehow challenging to control a disease with high transmissibility like COVID-19. Strict measures that aim to restrict these events and limit the possibility to communicate the disease within the society have been implemented (12, 17).

These measures included strict rules that banned all the following until further notice: social events and public gatherings, such as weddings and funerals, prayer's attendance at all mosques and churches, and social visits to hospitals and prisons (17). In addition, all sports facilities, cinemas, and youth centers were banned, as was shisha (Hookah) at cafes and restaurants, and restaurants and cafes were obligated to keep enough social distance between seats (12, 17).

Surprisingly, these measures were intensified on the 17th of March 2020 to include a strict ban on public gatherings of more than 10 persons, ban on inter-city travel and all public transportation, and closing all malls and commercial centers (12, 17). Then, on the 20th of March 2020, a country curfew was declared with a strict ban on individuals' mobility (17). Since declaring the curfew, the Jordanian government has actively worked to ensure compliance with rules and directions of the curfew and has taken multiple measures to facilitate and ease the movement of individuals for the acquisition of supplies for basic needs during total and partial curfew times (17). Various efforts by different authorities have been made to reduce the stress and increase the societal adaptability to the curfew.

### Overview of Decisions Made for the Public and Private Sectors

Many decisions that control different industries in Jordan have been made in order to protect the employees and their families. Although governmental and private institutions continued to work as usual until the middle of March, a critical decision was announced on the 17th of March 2020 that suspended all work duties at public and private sectors, with the closure of all industrial activities until further notice excluding vital industries, such as healthcare, energy, food, as well as the crisis task force (17). In addition, electronic platforms were created to gather information about vulnerable workers and families in order to support them financially through official channels (17).

Healthcare institutions and healthcare workers were exempted from the curfew rules in order to keep healthcare facilities functioning and ready for patients, taking into consideration the careful use of personal protective measures (12, 17).

### Overview of Decisions on Education

All academic institutions at all levels were closed effective of the 15th of March 2020 and until further notice. Accordingly, all teaching and learning activities moved toward distance learning platforms (17).

## COVID-19 Statistics in Jordan

**Chart 1:**
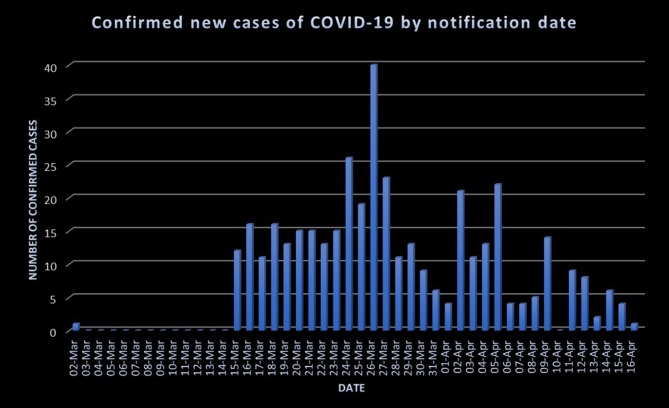
The number of new confirmed cases of COVID-19 by notification date during the period between the 2nd of March and the 16th of April 2020. Developed according to publicly declared statistics on the Jordanian COVID-19 website (12).

**Chart 2:**
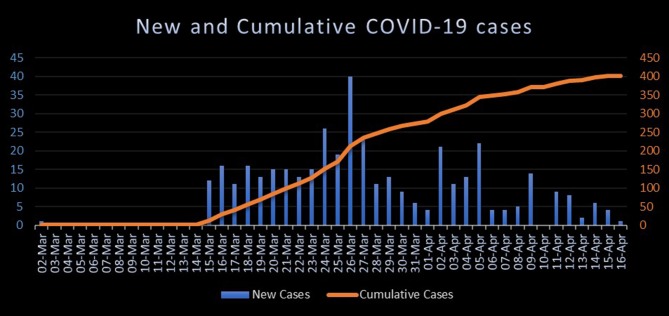
The number of new and cumulative cases of COVID-19 by date during the period between the 2nd of March and the 16th of April 2020. Developed according to publicly declared statistics on the Jordanian COVID-19 website (12).

The statistics about COVID-19 in Jordan are publicly announced by government officials and are available on a specific COVID-19 website created for this purpose, though the publicly announced statistics do not include any sensitive information about the patients (12). The first confirmed case of COVID-19 was registered in Jordan on the 2nd of March 2020: a young Jordanian male who was on a trip to Italy. Upon confirming the first case, the national measures were scaled up in order to limit and tackle the spread of COVID-19 effectively. As of the 16th of April 2020, there have been 402 confirmed cases of COVID-19 and seven deaths attributed to the disease (12, 17). In addition, most of confirmed cases were Jordanian nationals. The seven deaths occurred in the period between the 28th of March and the 9th of April 2020 for people of older age groups with underlying comorbidities as per the government officials (12). More details about these statistics are provided in Charts 1, 2.

## Discussion

In the previous decades, many emerging respiratory viruses and respiratory diseases have posed a threat to humans globally (19). It is important to focus on the value of having a national preparedness plan in response to emerging communicable diseases. In addition, lessons should be learned from the previous outbreaks and pandemics (20). The COVID-19 pandemic is an emerging public health issue that threatens human life and is an unpredictable situation with many uncertainties, thus exhibiting the main characteristics of a “crisis” (21, 22). Crises management is challenging to both policy makers as well as decision makers; an improper and incomplete response can lead to devastating outcomes (22).

This article has provided a brief overview of the ongoing Jordanian experience and response in combating COVID-19. The measures that were implemented by the government aimed particularly to mitigate the spread of the disease and to increase the societal awareness about this pandemic. From the previous charts, spikes in the number of new cases were noticed during the last week of March and the first week of April even though the country's lockdown and curfew preceded these spikes. This raises concerns about the behavior of SARS-CoV-2 and transmission dynamics.

Proper communication and information dissemination are essential in crises management (22). The Jordanian government has implemented various measures that are aimed at providing the public with essential information and directions by reaching different age groups across the country through media channels, such as television, Internet, and COVID-19 emergency hotlines, as well as through the armed and security forces who provided support and assistance for the public. Google™ has created a platform that collects and aggregates anonymous data on trends of individuals' mobility within the community across different countries using data from Google maps, aiming to support health officials and policy makers during this pandemic (23). As of the 11th of April 2020, Google mobility charts showed that individuals' mobility in Jordan has been effectively reduced during the curfew. Interestingly, the mobility around highly crowded spots was reduced: retail and recreation centers have seen a reduction by 93% compared to the baseline, grocery stores have seen a reduction by 89% compared to the baseline, and workplaces have seen a reduction by 81% compared to the baseline (23). The data from these mobility charts show that the governmental restrictions on individual's movements were effective and successful despite the few hundreds of violations that happened at the beginning of the curfew (24).

The psychological impacts associated with curfew and lockdown are also challenging to the society and the government. The extent of societal adaptability to this sudden change in lifestyles could be determined by the level of awareness among individuals and the degree of the governmental restrictions (25). Despite the limited number of violations that happened during the curfew, the Jordanian public showed high levels of commitment and awareness, as reflected by the slow pace of COVID-19 spread inside the country, and this implies that majority of the public have adopted the recommended preventive and control measures successfully. Different societal responsibilities, including social distancing, frequent hand washing and sanitization, as well as complying with the recommendations from health authorities, will all result in a more effective national response to limit the spread of the disease, especially upon the release of the current lockdown and curfew in Jordan.

The main goal of the lockdown and curfew strategy is preventing the exponential rise in the number of infected persons within a short period to avoid overwhelming the healthcare facilities (26). However, the process of returning to normal life after releasing the lockdown and curfew is also challenging to both decision makers and the society. Early reduction and easing up of governmental interventions and restrictions might lead to adverse impacts in causing a subsequent strike of COVID-19 (27). Looking at the fact that effective antiviral medications and vaccines are still lacking, the Jordanian decision makers should not ignore the possible scenario of a serious subsequent strike with COVID-19 cases after ending the current lockdown and curfew considering the slow pace of COVID-19 spread and the undeveloped population-scale immunity. Thus, plans for managing a possible “second wave” of infections must be incorporated into the lockdown and curfew exit strategy (26), and this should be supported by simulations of the effects of different public health measures to predict future scenarios (27). Bearing that in mind, the Jordanian public have a tremendous responsibility in terms of adapting to the COVID-19 preventive measures and implementing them as a new normal lifestyle, especially in the period following the lockdown and curfew.

Furthermore, the Jordanian preparedness and response strategy can benefit from the ongoing global experiences and scenarios regarding the COVID-19 pandemic. Although Jordan was among the first countries to implement highly strict preventive and control measures, there are always opportunities to learn from the global experience to improve the current national strategy. At this early stage and the uncertain future scenarios, it is difficult to critically compare the effectiveness of various COVID-19 response strategies at different contexts despite the fact that the numbers of COVID-19 cases and deaths in Jordan are much lower than in most of the neighboring countries. However, during and after the battle of COVID-19, countries, including Jordan, must take more serious steps to strengthen and improve their healthcare system capacity in order to be well-prepared for such crises in the future (28, 29). Having a sufficient reservoir of medical devices and personal protective equipment as well as a backup of highly trained healthcare staff for critical units will be of great assistance and support to keep going during pandemics.

As a country of limited resources, the COVID-19 pandemic is expected to have a noticeable negative impact on the Jordanian economy due to the ongoing country's lockdown and curfew. In response to that, an emergency response fund, with generous contributions from different components of the Jordanian public, has been created in order to reduce the economic impacts of this crisis (12, 17). Recently, the government also began to relax some restrictions by allowing certain commercial sectors to return to work under specific regulations (17). However, the individuals' commitment to and compliance with the preventive measures are critical during these relaxations. Besides, it is beneficial to carry out economic studies to develop some insight into the current economic status as well as the period that will follow the release of the lockdown and curfew.

In conclusion, the Jordanian way of combating the COVID-19 pandemic is promising despite the uncertain future predictions and scenarios. In addition, the Jordanian crises management task force provides an example of how important the collaborative efforts in providing critical decisions are. Adopting and implementing the technical guidelines in emergency health situations provided by the WHO is also crucial (30). Moreover, maintaining high levels of awareness and commitment within the Jordanian society, strengthening the government–society partnerships, having a well-formulated national preparedness and response strategy with effective leadership, as well as implementing internationally standardized guidelines in crises management are all essential to success and progress during critical situations like the COVID-19 pandemic.

## Data Availability Statement

The numbers of COVID-19 cases and deaths that were used to develop the charts for this study can be found on [https://corona.moh.gov.jo/].

## Author Contributions

The author confirms being the sole contributor of this work and has approved it for publication.

## Conflict of Interest

The author declares that the research was conducted in the absence of any commercial or financial relationships that could be construed as a potential conflict of interest.
